# The Potential of a Novel Class of EPAC-Selective Agonists to Combat Cardiovascular Inflammation

**DOI:** 10.3390/jcdd4040022

**Published:** 2017-12-05

**Authors:** Graeme Barker, Euan Parnell, Boy van Basten, Hanna Buist, David R. Adams, Stephen J. Yarwood

**Affiliations:** 1 Institute of Chemical Sciences, Heriot-Watt University, Edinburgh EH14 4AS, UK; Graeme.Barker@hw.ac.uk (G.B.); D.R.Adams@hw.ac.uk (D.R.A.); 2 Department of Physiology, Feinberg School of Medicine, Northwestern University, Chicago, IL 60611, USA; euan.parnell@northwestern.edu; 3 Institute of Biological Chemistry, Biophysics and Bioengineering, Heriot-Watt University, Edinburgh EH14 4AS, UK; bv9@hw.ac.uk (B.v.B.); hkb1@hw.ac.uk (H.B.)

**Keywords:** EPAC1, cyclic AMP, cyclic nucleotide binding domain, inflammation, endothelial cells, high-throughput screening

## Abstract

The cyclic 3′,5′-adenosine monophosphate (cAMP) sensor enzyme, EPAC1, is a candidate drug target in vascular endothelial cells (VECs) due to its ability to attenuate proinflammatory cytokine signalling normally associated with cardiovascular diseases (CVDs), including atherosclerosis. This is through the EPAC1-dependent induction of the suppressor of cytokine signalling gene, SOCS3, which targets inflammatory signalling proteins for ubiquitinylation and destruction by the proteosome. Given this important role for the EPAC1/SOCS3 signalling axis, we have used high throughput screening (HTS) to identify small molecule EPAC1 regulators and have recently isolated the first known non-cyclic nucleotide (NCN) EPAC1 agonist, I942. I942 therefore represents the first in class, isoform selective EPAC1 activator, with the potential to suppress pro-inflammatory cytokine signalling with a reduced risk of side effects associated with general cAMP-elevating agents that activate multiple response pathways. The development of augmented I942 analogues may therefore provide improved research tools to validate EPAC1 as a potential therapeutic target for the treatment of chronic inflammation associated with deadly CVDs.

## 1. Introduction

Cyclic adenosine monophosphate (cyclic AMP) is the prototypical second messenger [1,2]; its intracellular concentration is governed by the relative expression and localization of enzymes responsible for its synthesis, adenylyl cyclases (ACs), and degradation, cyclic AMP phosphodiesterases (PDEs) [3,4,5,6,7,8]. Cyclic AMP exerts most of its effects through the activation of a range of down-stream sensors, including protein kinase A (PKA) [9,10,11,12], exchange protein directly activated by cyclic AMP proteins (EPAC) [13,14,15], Popeye domain-containing (POPDC) proteins [16,17] and cyclic nucleotide gated (CNG) ion channels [18]. Due to the diverse physiological responses controlled by cyclic AMP, signalling drugs have been developed to either promote cyclic AMP production, through activation of ACs [19], or inhibit its breakdown through inhibition of PDEs [7,20,21,22,23]. Both of these strategies lead to elevations in intracellular cyclic AMP, with the potential to activate all PKA, EPAC, POPDC and CNG signalling routes, depending on cell type [24]. However, such indiscriminate activation may be problematic. For example, PDE inhibitors, such as pentoxifylline, ibudilast, drotaverine and roflumilast, can cause undesirable physiological effects, including nausea, emesis, diarrhoea and cardiac arrhythmia [25], limiting their therapeutic usefulness. Similarly, the use of the di-terpene, forskolin, to activate ACs, has also been linked to various side effects, including flush syndrome and hypotension [26]. Therefore, the strategy of promoting the activation of all cyclic AMP signalling routes (e.g., PKA, EPAC, POPDC and CNG) with PDE inhibitors and forskolin may be unsuitable in therapeutic scenarios and new approaches to reduce side effects should be considered. In this regard, it may be possible to develop compounds that selectively activate EPACs, while avoiding many of the side effects associated with global cyclic AMP elevation. The focus of this review will therefore concentrate on the potential benefits of selective EPAC activation for the treatment of vascular inflammation and efforts to produce small molecule EPAC agonists to achieve this goal.

## 2. EPAC Proteins

**Figure 1:**
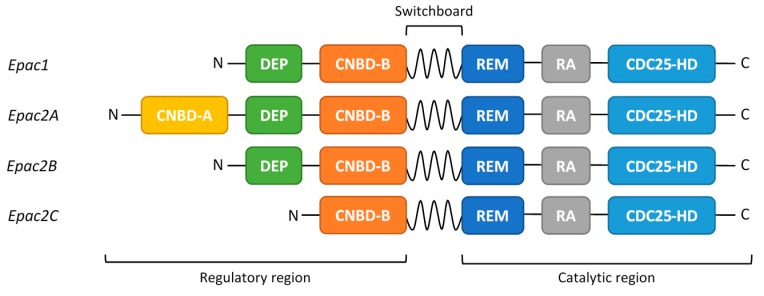
Primary structure of the different exchange proteins activated by cyclic AMP (EPAC) isoforms. The N-terminal regulatory region is directly connected to the C-terminal catalytic region through the switchboard region. Cyclic AMP interacts with the cyclic nucleotide-binding domain (CNBD-B), present in all EPAC isoforms, to trigger enzyme activation. Epac2A has an extra, non-functional cyclic AMP binding domain (CNBD-A). The other functional EPAC domains are indicated; DEP—Dishevelled, Egl-10, Pleckstrin domain, required for protein-protein and protein-lipid interactions; REM—Ras exchange motif, required for the stability of the CDC25-HD catalytic domain; RA—Ras association domain; allows interaction with members of the Ras-superfamily of small GTPases; CDC25-HD—CDC25 homology domain, which contains catalytic GEF activity to Rap 1/2 [14,36,37].

**Figure 2:**
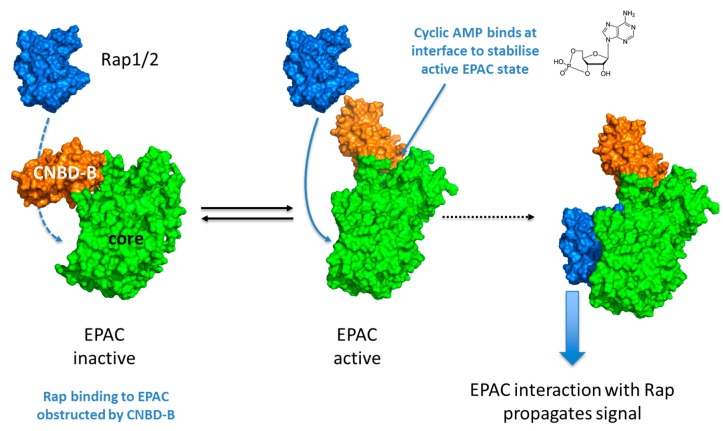
Activation of EPAC enzymes involves a conformational change triggered by the interaction of cyclic AMP with the EPAC CNBD-B.

EPAC proteins are cyclic AMP-regulated guanine nucleotide exchange factors (GEFs) that activate the small GTPases, Rap1 and Rap2 [27,28]. There are two main paralogues of EPAC, EPAC1 and EPAC2A [27,28], derived from distinct genes, in addition to two EPAC2A splice variants, EPAC2B and EPAC2C, which arise from differential promoter usage [29]. All EPAC isoforms consist of an N-terminal regulatory region and a C-terminal catalytic region (Figure 1) [29,30,31,32]. It is the binding of cyclic AMP to the regulatory cyclic nucleotide-binding domain (CNBD-B; Figure 1) that promotes GEF activity toward RAP1/2 [30,31,32] (Figure 2). The EPAC2A isoform contains an additional, N-terminal CNBD (CNBD-A; Figure 1), albeit with a much lower affinity for cyclic AMP [27,28]. It is not yet clear what the function of the second cyclic AMP binding domain is, although it has been speculated that the subcellular localization of Epac2A is, at least partly, regulated by the presence of this CNBD [33]. Other than this difference, EPAC1 and EPAC2 share similar structural motifs throughout the regulatory and catalytic domains. Indeed, the dishevelled-EGL-pleckstrin homology domain (DEP), Ras exchange motif (REM), Ras association domain (RA) and CDC25 homology GEF domains are conserved between isoforms (Figure 1) [34,35,36]. In the absence of cyclic AMP, EPAC is held in an inactive conformation due to intramolecular interactions between the regulatory CNBD-B and the catalytic GEF domain (Figure 2) [30,31]. Cyclic AMP binding to the phosphate binding cassette of the CNBD-B results in a local tightening and closure of the “lid” region over the cyclic AMP binding pocket (Figure 2) [32]. The conformational changes induced by binding cyclic AMP evoke an open form of EPAC that allows the GEF domain to interact with and activate Rap1 and Rap2 (Figure 2) [30,31].

## 3. EPAC1 Signalling and Vascular Function

A number of studies have suggested that EPAC-selective ligands may be useful for the future treatment of cardiac arrhythmia [38], obesity [39,40], diabetes [41], hypertension [42], cancer [43] and inflammatory pain [44]. Concerning inflammation, it has been suggested that selective EPAC regulators may be useful for the treatment of IL-8 driven lung inflammation associated with chronic obstructive pulmonary disorder (COPD), where EPAC2 appears to be pro-inflammatory, whereas EPAC1 suppresses lung remodelling [45,46,47]. Moreover, evidence is also emerging that EPAC1 is also a candidate drug target in vascular endothelial cells (VECs) due to its ability to attenuate pro-inflammatory cytokine signalling normally associated with atherosclerosis and neointimal hyperplasia (NIH), which arises from mechanical injury during angioplasty with stents [14,37]. This is because the VEC layer provides an important barrier to circulating inflammatory cytokines and leukocytes, and damage or disruption to this barrier is a key etiological precursor to various cardiovascular diseases. In addition, NIH is characterised by localised inflammation and proliferation of vascular smooth muscle cells (VSMCs) that underlie the VEC layer, thereby precipitating stent failure and myocardial infarction [48]. EPAC1 has been shown to inhibit migration of VSMCs associated with NIH [49,50], although a number of conflicting reports have been published (Table 1) and further work needs to be done in this area. Despite this, EPAC1 has emerged as an important factor in the regulation of the pro-inflammatory interleukin 6 (IL-6) trans-signalling pathway in VECs [51]. This makes EPAC1 an interesting candidate therapeutic target for the treatment of diseases in which IL-6 signalling is heavily implicated, such as atherosclerosis [52,53,54,55,56].

### 3.1. IL-6 Signalling in Vascular Endothelial Cells

**Figure 3:**
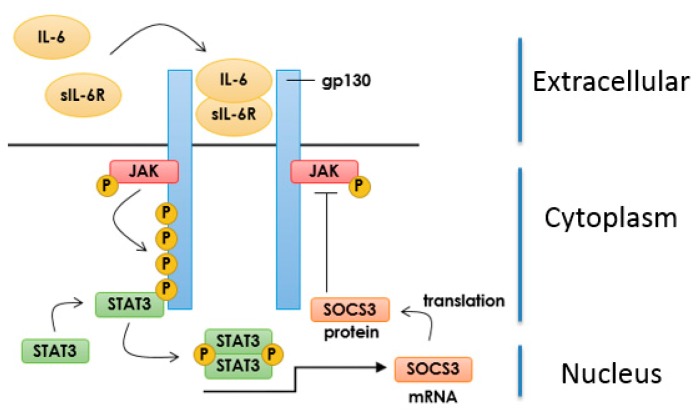
IL-6 Signalling and SOCS3 Induction. IL-6 binds to the soluble form of the IL-6 receptor (sIL-6R) thereby promoting the dimerization of gp130 glycoprotein and activation of receptor-associated JAKs. Activated JAKs phosphorylate STAT3, leading to its dimerization and translocation to the nucleus where it initiates gene transcription including induction of the suppressor of cytokine signalling (SOCS) 3 gene. SOCS3 protein then serves as a negative feedback regulator of JAK-STAT signalling.

IL-6 signalling is mediated by a receptor complex comprising an α chain (IL-6Rα) and a transmembrane glycoprotein (gp130) that associate on the cell surface in the presence of the cytokine to form a 2:2:2 heteromeric complex [57]. However, the pathophysiological pro-inflammatory actions of IL-6 in a variety of diseases, including atherosclerosis [56], are thought to be driven by aberrant IL-6 receptor “trans-signalling” [53]. During trans-signalling, IL-6 binds to a soluble form of the IL-6R (sIL-6R) and this then allows IL-6 to activate gp130 on the surface of cells that are normally unresponsive to IL-6, including VECs [53]. The new signalling complex, formed of IL-6/sIL-6R/gp130, can now activate Janus tyrosine kinases (JAKs) in VECs, which then phosphorylate the cytosolic region of gp130 on key residues, including Tyr767, Tyr814, Tyr905 and Tyr915 [58]. Phosphorylation of these sites leads to the recruitment, JAK-dependent phosphorylation and activation of signal transducer and activator of transcription 3 (STAT3), which dimerizes and translocates to the nucleus (Figure 3), where it promotes transcription of pro-inflammatory genes [59], including VEGF and MCP-1. 

If unresolved, IL-6 trans-signalling will maintain an inflammatory condition by promoting the recruitment and activation of inflammatory cells, endothelial dysfunction and promoting VSMC proliferation and migration [64].

Normally, IL-6 signalling is controlled through a classical negative-feedback route involving the induction of the gene encoding suppressor of cytokine signalling protein (SOCS) 3 by the same JAK-STAT3 pathways that the IL-6R activates [65] (Figure 3). Once induced SOCS3 acts by binding to JAK-phosphorylated receptors, mediated by the SOCS3 SH2 domain, thereby inhibiting JAK activity, STAT signalling [66] and targeting JAK for proteasomal degradation [67].

### 3.2. Inhibition of IL-6 Signalling by SOCS3

SOCS3 is a potent inhibitor of pro-inflammatory pathways involved in atherosclerosis [68] and the development of NIH [69]. Indeed, SOCS3 expression is increased in atherosclerotic plaques [70,71] and its knockdown in apoE −/− mice increases inflammatory gene expression in aorta, leading to enhanced atherogenesis [71]. Moreover, knockdown of SOCS3 promotes pro-inflammatory actions of IL-6 [72] and triggers angiogenesis in VECs [73]. In contrast, overexpression of SOCS3 suppresses JAK/STAT signalling and the development of atherosclerosis and NIH, demonstrating the importance of SOCS3 in limiting the development of cardiovascular disease [69,74,75].

### 3.3. Induction of SOCS3 by EPAC1 and Inhibition of IL-6 Signalling in VECs

Activation of EPAC1 in VECs leads to a down-regulation of IL-6-mediated inflammatory processes through the JAK/STAT3 pathway [51], which occurs through C/EBP transcription factor-dependent SOCS3 induction [76]. Moreover, EPAC1 exerts other cyclic AMP-dependent anti-inflammatory actions in VECs, including activation of integrins, thereby promoting adhesion of VECs to the basement membrane [77]. In addition, EPAC1 activation is able to promote endothelial barrier function [78,79] through VE-cadherin mediated cell-cell junction stability [80], in response to actin [81,82,83,84,85] and microtubule [86] cytoskeletal reorganisation. Thus, EPAC1 is involved in multiple anti-inflammatory processes in VECs and the links between EPAC1 and SOCS3 and the development of atherosclerosis/NIH indicate that EPAC1 regulation should be considered as a potential therapeutic avenue for the future treatment of cardiovascular disease.

## 4. Development of EPAC-Selective Agonists

### 4.1. EPAC Agonists Based on Cyclic Nucleotides

**Figure 4:**
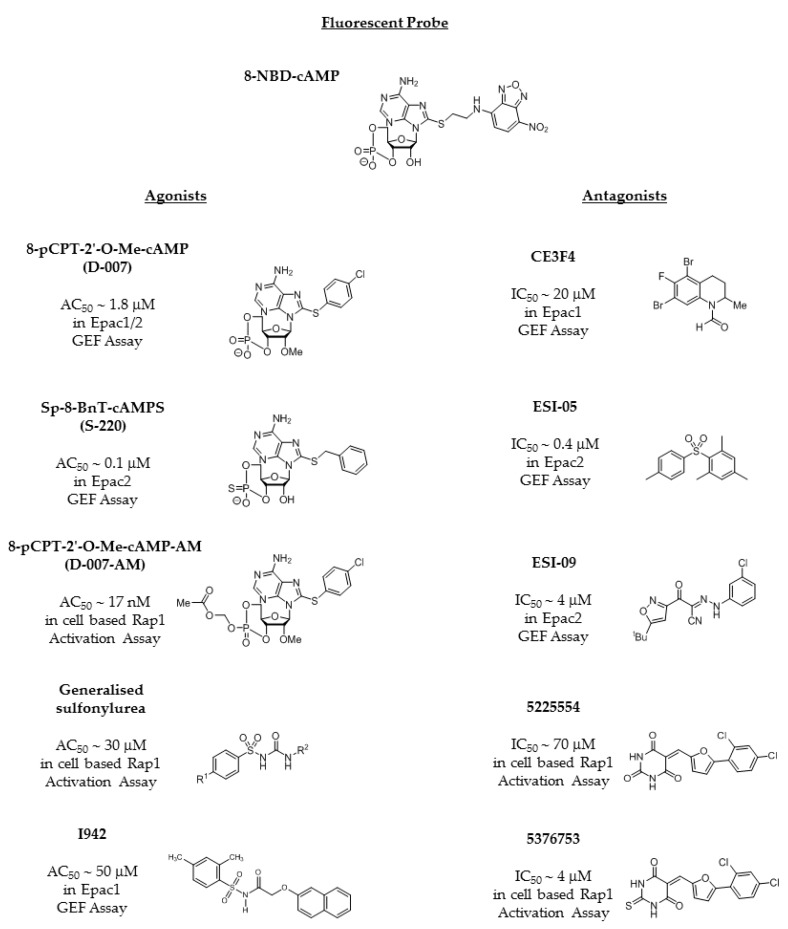
Chemical structures of existing EPAC-selective agonists and antagonists together with the cyclic analogue AMP analogue fluorescent probe molecule, 8NBD-cAMP.

Due to the emerging potential of EPAC isoforms as drug targets, efforts have been made to develop small molecule EPAC agonists. The first of these, the cyclic AMP analogue, 8-(4-chlorophenylthio)-2′-*O*-methyladenosine-3′,5′-cyclic monophosphate (8-pCPT-2′-*O*-Me-cyclic AMP; 007), and its improved cell permeable derivative (007-AM [87]; Figure 4), are able to selectively activate both EPAC1 and EPAC2 isoforms, independently of PKA [88]. The selectivity of 007 for the EPAC isoforms over PKA is due to a single amino acid difference in the CNBD cyclic AMP-binding pockets of PKA and EPACs, which are otherwise highly conserved in their amino acid composition [88]. Thus, the substitution of a key glutamic acid residue in the PKA CNBD, by a glutamine or lysine in EPAC1 or EPAC2, respectively, is responsible for the discrimination exhibited by the 2′-*O*-methylated nucleotide and preference for binding to the EPACs [88]. A structural understanding for the basis of this selectivity was assisted by the determination of the 3D structure of EPAC2 [30,31], and now a range of cyclic AMP analogues with varying kinetic properties have been developed that can also differentiate between EPAC1 and EPAC2 [89]. In particular, an EPAC2-selective agonist, 8-benzylthioadenosine-3′,5′-cyclic monophosphorothioate (Sp-8-BnT-cAMPS; S-220; Figure 4), has been developed that exerts glucose-dependent stimulatory activity in insulin-secreting human pancreatic cells [90]. However, in vivo use of these analogues has been hampered by cardiac arrhythmia, fibrosis and cardiac hypertrophy in animal models [91,92], limiting rigorous preclinical assessment of their therapeutic benefit. These effects are likely linked to calcium signalling crosstalk within cardiomyocytes [93,94] following chronic activation of EPAC2 within the heart. These observations suggest that pharmacological exploitation of EPAC-activating compounds may need to be focused on the development of selective EPAC1 ligands, thereby avoiding these adverse effects.

### 4.2. The Sulfonyl Urea Family as EPAC Agonists

After 007 and its analogues, the most studied, but controversial, group of small molecule EPAC agonists are those of the sulfonylurea (SU) family (Figure 4). SUs were originally developed as anti-diabetic drugs capable of regulating the SUR1 receptor, leading to the opening of ATP-dependent potassium channels in pancreatic β-cells, with consequent calcium release and increased insulin exocytosis [95]. Recently it has been postulated that SUs also act as isoform selective activators of EPAC2, in particular via an allosteric mechanism involving the low affinity CNBD-A of EPAC2 [96]. This idea has been challenged [97], however, and it has been shown that SUs are unable to induce GEF activity in in vitro EPAC2 activation assays [98]. Insulin secretion is known to be impaired in EPAC2 knockout mice, but the positive effect of SUs on EPAC2 activity in cellular assays may be indirect, as reports indicate that these compounds induce elevations in intracellular cyclic AMP [98].

### 4.3. EPAC Antagonists

Further efforts to identify small molecule EPAC regulators using high throughput screening (HTS) of compound libraries have mainly identified antagonists, both orthosteric and allosteric, rather than agonists [1,2,99,100]. Indeed, uncompetitive (CE3F4) and non-competitive EPAC1 inhibitors (5225554 and 5376753; Figure 4) have been identified using in vitro EPAC1 GEF [2] and EPAC-based bioluminescence resonance energy transfer-based [1] assays, respectively. Similarly, inhibitors for EPAC2 have been identified by HTS using a displacement assay with the cyclic AMP analogue, 8-[2-[(7-nitro-4-benzofurazanyl)aminoethyl]thio]-cyclic AMP (8-NBD-cyclic AMP; Figure 4), which fluoresces when bound within the hydrophobic environment of the cyclic AMP binding pocket [89]. Displacement of 8-NBD-cyclic AMP by competitor compounds leads to a reduction in fluorescence, allowing the identification of interacting molecules. Despite this assay being equally sensitive to agonist and antagonist molecules, so far only the EPAC2-selective competitive inhibitor, ESI-05 (4-methylphenyl-2,4,6-trimethylphenylsulfone) [101], and a non-selective EPAC1/2 inhibitor, ESI-09 (3-(5-*tert*-butylisoxazol-3-yl)-2-[(3-chlorophenyl)-hydrazonol]-3-oxopropionitrile), have been identified [99,102,103]. Subsequently, concerns were raised over the specificity of ESI-09, which is thought to display non-specific protein denaturing properties [104]. Despite this, several more potent EPAC antagonistic ESI-09 and ESI-05 analogues (Figure 4) have been developed [105,106,107].

### 4.4. Identification of Non-Cyclic Nucleotide (NCN) EPAC Agonists

HTS using 8-NBD-cAMP (Figure 4) competition assays has been limited to screens involving EPAC2, likely due to the limited fluorescence of 8-NBD-cAMP when bound to the CNBD-B of EPAC1 compared to EPAC2 [99]. This difference may be linked to structural differences between EPAC1 and EPAC2 within the CNBD-B that selectively influence the docking of ligands to the cAMP-binding pocket [90]. Moreover, the low stability of full length recombinant EPAC1 in vitro has limited its study in HTS and structural assays [89]. Although full-length EPAC1 is relatively unstable in vitro, the isolated EPAC1-CNBD-B displays superior stability and 8-NBD-cAMP has been shown to bind and fluoresce within the CNBD-B of EPAC1 [89]. Given that the CNBDs of EPAC1 and EPAC2 display structural differences [108,109,110], screening both CNBDs simultaneously may facilitate isoform-selective compound discovery. We have used this dual-target strategy to screen 5195 compounds from the BioAscent Compound Collection (Biocity Scotland, Newhouse, Scotland), identifying a number of ligands with varied binding affinities to the distinct isoforms [111]. Follow up characterisation of the top hit, I942 (Figure 4), using ligand observe nuclear magnetic resonance (NMR) confirmed direct interaction with both EPAC1 and EPAC2 CNBD-B [111]. However, in vitro GEF assays revealed that I942 displayed partial agonist activity toward EPAC1 (AC_50_ 10% that of cyclic AMP [111]) and no significant action towards EPAC2. Further study revealed that I942 had no effect in vitro on PKA activity as measured by phosphorylation of the transcription factor CREB, a known PKA substrate. I942 is therefore the first non-cyclic nucleotide small-molecule with selective agonist properties toward EPAC1. As a novel, NCN scaffold, the *N*-acylsulfonamide chemotype of I942 might be advantageous to nucleotide-based structures, which may require prodrug strategies for therapeutic deployment. 

### 4.5. Putative Binding Mode of Novel EPAC Agonists

**Figure 5:**
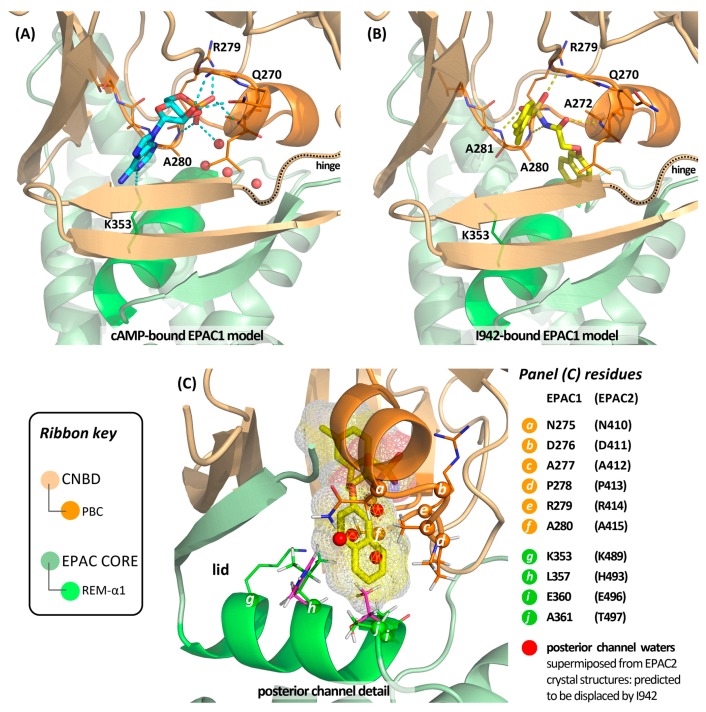
Modelling studies with EPAC1 homology models suggest that I942 may engage the cyclic nucleotide binding domain-B (CNBD-B) with subsequent hinged closure of the domain onto the protein core leading to a “threaded” binding mode and adoption of an active-state EPAC conformation. In this binding mode (panel (**B**)) the acidic *N*-acylsulfonamide is predicted to occupy the position of the endogenous ligand’s phosphate (panel (**A**)) and form extensive phosphomimetic hydrogen bonds (dashed lines) with the protein’s phosphate-binding cassette (PBC, bright orange ribbon). EPAC2 co-crystal structures show that the cyclic AMP adenine binds in a large funnel-like channel that opens on the front face of the protein as figured in panel (**A**), where the adenine N1 centre engages a lysine (conserved as K353 in EPAC1) on the REM domain’s helix-α1 (bright green ribbon). An additional smaller opening, the posterior channel, communicates to the reverse face of the protein. Cyclic AMP does not directly utilise this volume, which is occupied by water molecules in available EPAC2 co-crystal structures (marked as red spheres in the panel (**A**) model). We postulate that this posterior channel, which is heavily hydrophobic, hosts the naphthyloxy group of I942, as detailed in panel (**C**). Occupancy of this channel may be entropically favoured by displacement of water (shown superimposed as red spheres in panel (**C**)), and stabilise closure of the CNBD onto the EPAC1 core by interaction of the naphthyl subunit with REM-α1 residues L357, E360 and A361 (*h*, *i* and *j* in panel (**C**)). L357 and A361 are not conserved across the EPAC isoforms, which may account for the observed selectivity of I942, as the cognate EPAC2 residues (H493, T497; magenta stick) are predicted to interdict I942 binding. In the EPAC active conformation the REM-α1 helix folds as a lid onto the ligand binding site due to reorganisation of the EPAC hinge sequence (dotted ribbon in panels (**A**,**B**)). Whilst the naphthyloxy group may favourably engage the surface of REM-α1 at the C-terminal end, it fails (in contrast to cyclic AMP) to engage K353 at the N-terminal end. This may influence equilibrium position between CNBD-B open and closed states, with weaker overall engagement of the REM-α1 lid by I942 (or/and ligand-specific domain seating penalties) accounting for the observed EPAC1 “partial agonism” relative to the endogenous ligand.

The binding mode of I942 and mechanism of EPAC1 activation remain to be experimentally determined, although in silico modelling studies have been undertaken and a plausible binding postulate developed to rationalise the compound’s partial activation of EPAC1 (Figure 5). As the structure of EPAC1 has yet to be determined, homology models of EPAC1 were constructed from crystal structures (PDB: 4MH0, 4MGY, 4MGK) of EPAC2 in the nucleotide-bound, active conformation with and without an EPAC1-mimetic point mutation (K405Q) [90]. These models allowed us to explore the possible structural basis for interaction of I942 with EPAC1. Preliminary findings suggest that the acidic *N*-acylsulfonamide motif (pKa ~ 4) may occupy a similar volume to the cyclic AMP phosphate (Figure 5), engaging a key charge-pairing arginine (Arg279) within the CNBD “phosphate-binding cassette” (PBC), defined by residues 268-FGQLALVNDAPRAAT-282 of EPAC1 [32]. The PBC is strongly conserved in EPAC2 as residues 403-FGKLALVNDAPRAAS-417 in the murine constructs used for crystallography, and contains a short helix that hydrogen bonds through its N-terminus to the phosphate of cyclic AMP. The ionised *N*-acylsulfonamide is similarly predicted to cap this helix, through its carbonyl oxygen, whilst additionally engaging the PBC backbone at Ala280 and Ala281 in charge-stabilised hydrogen bonds from the nitrogen and one of the sulfonyl oxygens. 

Our binding hypothesis positions the I942 *m*-xylyl group approximately coplanar with the bound nucleotide’s purine in the main funnel-like opening to the binding site. However, direct overlap with the adenine bicycle is limited in this model, and I942 does not exploit the polar interactions available to the endogenous ligand through the adenine bicycle. Thus, co-crystal structures of EPAC2 constructs with bound cAMP reveal that a key lysine (Lys489) on helix-α1 of the REM domain engages the purine N-1 centre. This promotes folding of the cyclic AMP-bound CNBD onto the REM domain surface, with the helix contributing to the EPAC “lid” region that closes over the nucleotide [30]. Lys489 is conserved on the REM-α1 helix of EPAC1 as Lys353, but I942 lacks the necessary structural extension and functionality to engage it. On the other hand, our model suggests that I942 may exploit additional, hydrophobic interactions at the opposite end of the REM-α1 helix to Lys353 that are not accessible to cyclic AMP. In particular, the model invokes threading of the oxymethylene linker through a narrow passage (solvent filled in the absence of ligand; Figure 5) that leads to a second and smaller funnel opening on the opposite face of the protein surface to the adenine-binding channel. It is this second “posterior channel”, we postulate, that hosts the I942 naphthyl moiety (Figure 5) and that (based on residue differences between EPAC2 and EPAC1) may be more restrictive in the case of EPAC2. 

The posterior channel is heavily hydrophobic, with the side chains of several conserved CNBD residues (Leu271, Asn275, Ala277, Pro278, Ala280 and Leu314) contributing much of the putative contact surface for the ligand’s naphthyloxy group. However, three residues from the REM-α1 helix of EPAC1 are also predicted to make a significant contribution to the posterior channel—namely Leu357, Ala361 and Glu360 (the latter through its side chain methylenes). Of these three residues, only the glutamic acid is conserved in EPAC2, with Leu357 and Ala361 replaced by histidine and threonine respectively. Our model suggests that packing of the napthyloxy group against these three REM-α1 residues may stabilise the closed, active state of EPAC1, albeit less effectively than cyclic AMP through its interactions in the anterior channel and perhaps with slightly altered seating of the CNBD against the EPAC core. This would account for the partial agonism, whilst the selectivity of I942 for activation of EPAC1 over EPAC2 may be explained, at least in part, by loss of the favourable surface contact with Leu357 and steric interdiction by the threonine replacement for Ala361. An implicit corollary of this “threaded model”, in which the ligand binds between anterior and posterior channels, is that the mechanism of EPAC1 activation must involve stepwise binding of the ligand to the open, inactive conformation of the protein followed by hinged closure of the ligand-bound CNBD (*cf*. Figure 2). Structural studies with EPAC2 have shown that the conformation of the hinge region is sensitive to a single point mutation in the PBC, where Lys405 of EPAC2 is replaced by a glutamine, which is located at the cognate position of EPAC1 (Gln270) [90]. At present, we cannot rule out the possibility that this difference between EPAC1 and EPAC2 might also contribute to the observed selectivity of I942 by differentially modulating the seating properties of the PBC against the lid for the two EPAC isoforms. However, the model presented in Figure 5 does not invoke a direct and EPAC1-specific contact between I942 and the side chain of Q270.

## 5. Conclusions

In summary, several cyclic nucleotide analogues have been developed as EPAC agonist tool compounds in recent years, some exhibiting discrimination between the two EPAC isoforms in addition to selectivity over PKA. To address the challenging physicochemical properties of nucleotides and enhance cell permeability, phosphate masking strategies have been used, as with the labile acetoxymethyl ester modification (007-AM; Figure 4) of the prototypical nucleotide agonist, 8-pCPT-2′-O-Me-cyclic AMP. Both nucleotide and non-nucleotide EPAC antagonists have also been reported. Very recently, we have identified a unique class of selective NCN EPAC1-activating ligand, exemplified by I942 (Figure 4). This new chemotype likely binds and stabilises the active state of EPAC1 via a previously unobserved interaction mode. Further work is required to validate the binding mode proposed for I942, but, if correct in its essentials, the model presented here suggests that there should be significant scope for optimisation of the ligand’s naphthyl and m-xylyl subunits to enhance affinity and adjust efficacy with respect to the GEF activity of EPAC1. Changes to the m-xylyl group might additionally be harnessed, in principle, to modulate the pKa of the *N*-acylsulfonamide, which is a pharmacologically well-precedented moiety [112] and which we suggest serves as a cyclophosphate ester mimetic in the case of I942. We therefore propose that an integrated programme of chemical synthesis of structural analogues with concurrent assessment of bioactivity may allow the generation of a “molecular toolkit” of ligands displaying a spectrum of activity from partial to full agonism and with prospects for tractable bioavailability. The development of such a toolkit will allow a full exploration of the roles of EPAC1 in VECs, as well as in preclinical disease models of vascular dysfunction. An experimental and comprehensive structure activity relationship (SAR) study of I942 alongside confirmation of our docking models may pave the way to the development of new therapies for the treatment of cardiovascular disease.

## Figures and Tables

**Table 1:** Effects of EPAC1 on experimental neointimal hyperplasia.

Experimental Model	Treatments	Effects
Carotid arteries and vascular smooth muscle cells (VSMCs) from wild type (WT) and EPAC1 −/− mice.	Ligation of carotid arteries and pharmacological inhibition of EPAC1	Neointima formation and VSMC proliferation were reduced in EPAC1 −/− mice. ESI09 also reduced neointima formation [60].
VSMCs from thoracic aorta explants from WT and EPAC1 −/− mice.	Injury of femoral artery	Reduced neointima formation and reduced migration of VSMCs in EPAC1 −/− mice [61].
Human saphenous vein VSMCs	Effects of pharmacological EPAC activation on VSMC migration	EPAC activation reduced VSMC migration and serum-induced vessel wall thickening [49].
Rat VSMCs from aorta explants.	Phamacological activation of EPAC and PKA	A combination of EPAC and PKA activation inhibited serum-induced VSMC proliferation [50].
VSMCs from foetal and adult rat aorta.	Pharmacological activation of EPAC and adenovirus-mediated overexpression of EPAC1	EPAC activation and overexpression of EPAC1 enhanced intimal thickening in aorta and VSMC proliferation [62].
Primary aortic VSMCs from male rats	Pharmacological activation of EPAC and PKA	PKA and EPAC work cooperatively to inhibit VSMC migration [63].
